# Meet the Editors‐in‐Chief

**DOI:** 10.1002/ansa.20190011

**Published:** 2020-04-07

**Authors:** Sebastiaan Eeltink, Paul Trevorrow

**Affiliations:** ^1^ Vrije Universiteit Brussel Brussels Belgium; ^2^ Executive Journals Editor Wiley Chichester UK


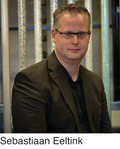


1

**Would you briefly explain what your research group is studying?**

The Eeltinklab at the Vrije Universiteit Brussel (Belgium) focuses on the design, development, and application of novel analytics for high‐resolution profiling of biomolecules and biomolecular interactions directly in complex life‐science matrices using ultra‐high‐pressure liquid chromatography (UHPLC) and multidimensional liquid‐chromatography (LC) workflows with mass spectrometry (MS) detection.

The focus is directed toward four research themes. (1) Advancing kinetic performance limits and detection sensitivity in UHPLC and LC×LC‐MS, hence we push the barriers with respect to realizing extremely fast (ballistic) separations and very high‐resolution separations. (ii) The development of novel column technologies including monolithic nanomaterials and characterization of emerging (commercially available) columns. The ability to control the external porosity and to tune the dimensions stationary phases on multiple length scales provides the possibility of tailoring the monolithic support structure toward separation performance. Complementary chromatographic approaches and physical characterization techniques are utilized to link column structure to separation performance. (iii) We pursue novel concepts via microfluidic solutions aiming at pushing performance limits in multidimensional 2D‐LC and 3D‐LC separations. (iv) Finally, we work on UHPLC‐MS and 2D‐LC‐MS method development in support of postgenomic biotechnology research and medical diagnostics.

**Why did you choose a career in separation sciences?**

I have always been passionate about analytical sciences. During a BSc research internship working on HPLC method development, I was supervised by Dr A. Tudos who kindly helped me with finding the best program to continue my MSc study. Here it was Prof R. Tijssen who sparked my interest in column technology in general and also the relation between column structure and resulting chromatographic performance limits. Column design and characterization is now one of the main research themes of my research group.

**Of all your research projects, which one was your favourite and why?**

In 2015, my team developed the first prototype chip ever for spatial three‐dimensional LC separations where components are separated in the space domain with each peak being characterized by its coordinates in a three‐dimensional (3D, microfluidic) separation body. While 3D‐LC has the potential to offer uniquely high resolving power within a short analysis time, the technology needs to be developed from scratch and many hurdles still need to be overcome.

 
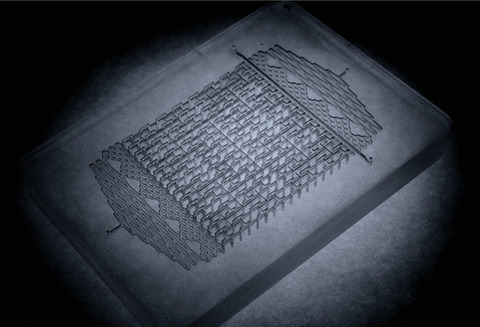


**What is your vision as editor on analytical science advances?**

I would like to create a publishing entity that collects research performed at the forefront of analytical sciences and allows peers to rapidly disseminate their research in a top‐notch journal. ASA will be open access, hence all barriers to access articles have been removed, which allows to elevate visibility of analytical chemistry among broad community of scientists working in different disciplines.

**Who were the most influential people in your career?**

I have been very fortunate with the support I have received from my supervisors (Dr W. Kok and Prof P. Schoenmakers) during my PhD study at the University of Amsterdam (Amsterdam, The Netherlands). Afterward, I moved to UC Berkeley (USA) where I was very fortunate to have the opportunity to work with Professor Frantisek Svec. I have received very good advice on many topics since but there are many more people that have and are contributing to my career.

The most influential people in my work may actually be the past and current members of the Eeltinklab. I have been very fortunate with so many nice researchers working dedicated and with great success on a variety of topics including monolithic capillary columns, UHPLC technology, and microfluidic chips. They contribute the most to my daily professional life and it has been and is a great a pleasure working with them.

**As a mentor and advisor, what do you advise your students in general?**

A PhD study is definitely challenging. In order to become successful, you need to work hard (together) and take responsibility. Furthermore, do not be afraid for making mistakes as this is part of the learning process. And most importantly, have fun in what you do!

**What do you consider to be the more exciting topics in analytical chemistry?**

One of the exciting topics is the miniaturization of (bio‐)analytical systems, that is, the development of sensors, system‐on‐a‐chip approaches, or even lab‐on‐chip devices. I believe that the development of integrated microfluidic and nanofluidic devices will play an essential role in, for example, clinical diagnostics and the next‐generation life science research.

An example of another interesting trend is the introduction of “Laboratory 4.0,” which addresses the growing complexity of (interdisciplinary) laboratory processes. Key innovation drivers include automation, robotics, and data management. But it will be very interesting to see how we build bridges between smart laboratories and life science research in the future.

**What are your views on the future of your field?**

Significant advances in analytical sciences and especially qualitative and quantitative chemical analysis (in all of its aspects including data analysis) are needed to address emerging problems and advance society in general. Miniaturization and development of integrated and portable measuring devices create new diagnostics possibilities, for example, for real time monitoring of (biological) processes, in ways that are currently are not even considered.

**What are your favourite past times outside of science?**

One of my passions is music, in particular playing percussion in orchestra. During my MSc study in Chemistry I have also followed a study “orchestral conducting” at the conservatory with the conductor Jan Schut. During the following years, I have conducted different orchestra. As a player and conductor, I was lucky to participate in a variety of momentous and breathtakingly beautiful concerts.

**What would you do if you had 1‐year paid leave?**

After taking some time for traveling I would love to spend time as visiting scientist working in academy and industry and get familiar in different disciplines related to life science research, that is, clinical diagnostics/single cell analysis, and biopharma/ biotechnology and bioprocessing.

**What nonscientist inspires you the most?**

I am continuously listening to music and exploring new songs. Hence instead of mentioning one or two names I would like to highlight all these artists who bring something beautiful to the world.

